# Protective Effects of *Atractylodis lancea* Rhizoma on Lipopolysaccharide-Induced Acute Lung Injury via TLR4/NF-κB and Keap1/Nrf2 Signaling Pathways In Vitro and In Vivo

**DOI:** 10.3390/ijms232416134

**Published:** 2022-12-17

**Authors:** Kun Shi, Yangxin Xiao, Yan Dong, Dongpeng Wang, Ying Xie, Jiyuan Tu, Kang Xu, Zhongshi Zhou, Guosheng Cao, Yanju Liu

**Affiliations:** 1 College of Pharmacy, Hubei University of Chinese Medicine, Wuhan 430065, China; 2 Center for Hubei TCM Processing Technology Engineering, Wuhan 430065, China

**Keywords:** acute lung injury, ethanolic extract of *Atractylodis rhizoma*, inflammatory response, oxidative stress

## Abstract

Acute lung injury (ALI) is a syndrome caused by an excessive inflammatory response characterized by intractable hypoxemia both inside and outside the lung, for which effective therapeutic drugs are lacking. *Atractylodis rhizoma*, a traditional Chinese medicine, has excellent anti-inflammatory and antiviral properties in addition to protecting the integrity of the cellular barrier. However, few studies of *Atractylodis rhizoma* for the treatment of ALI have been published, and its mechanism of action remains unclear. In the present study, the chemical composition of the ethanolic extract of *Atractylodis rhizoma* (EEAR) was initially clarified by high performance liquid chromatography (HPLC), after which it was studied in vivo using a lipopolysaccharide (LPS)-induced ALI rat model. Treatment with EEAR significantly reduced the lung wet/dry (*W*/*D*) ratio, neutrophil infiltration, and malondialdehyde (MDA) and myeloperoxidase (MPO) formation, and enhanced superoxide dismutase (SOD) and glutathione (GSH) depletion in rats with ALI, thereby improving lung barrier function and effectively reducing lung injury. In addition, EEAR significantly reduced histopathological changes, decreased the expression of inflammatory factors (such as tumor necrosis factor-α (TNF-α), interleukin-1 beta (IL-1β), inducible nitric oxide synthase (INOS), and cyclooxygenase-2 (COX-2)), and inhibited the activation of the NF-κB signaling pathway, thus reducing inflammation. In addition, EEAR was found to also reduce oxidative stress in ALI by upregulating the expression of nuclear factor erythroid 2-related factor 2 (Nrf2) and its downstream proteins heme oxygenase-1 (HO-1) and NADPH quinone acceptor oxidoreductase 1 (NQO-1). EEAR also reduced LPS-induced inflammatory factor expression in THP-1 cells in vitro by inhibition of the NF-κB signaling pathway, and reduced damage from lipopolysaccharide (LPS)-induced oxidative stress in THP-1 cells by promoting the expression of Nrf2 and its downstream targets HO-1 and NQO-1, the molecular mechanism of which was consistent with in vivo observations. Therefore, we conclude that EEAR attenuates oxidative stress and inflammatory responses via TLR4/NF-κB and Keap1/Nrf2 signaling pathways to alleviate LPS-induced ALI, suggesting that *Atractylodis rhizoma* is a potential drug candidate for the treatment of ALI.

## 1. Introduction

Acute lung injury (ALI) is a clinical syndrome characterized by intractable hypoxemia due to various intra- and extra-pulmonary pathogenic factors, causing acute diffuse lung injury leading to acute respiratory failure, the pathogenesis of which has not yet been fully elucidated. The pathology of ALI is characterized by alveolar inflammatory exudate and increased pulmonary vascular permeability causing pulmonary edema, leading to impaired gas exchange and acute deterioration [1]. Although ALI has been extensively studied over several decades, an effective treatment has not yet been developed. Current treatment options mostly reduce inflammation and suppress respiratory failure using drugs such as dexamethasone, prednisolone, and prednisone which can cause various adverse reactions, including coagulation disorders, gastric ulcers, or osteoporosis [2,3,4].

It has been demonstrated that ALI has two principal causal factors. The endotoxin released by bacterial lysis after lung infection, lipopolysaccharide (LPS), is one such factor, the exposure of which to the lungs causes the activation of macrophages and infiltration of inflammatory cells, leading to oxidative stress and an inflammatory response [5,6,7]. Secondly, sepsis, which can directly cause lung injury through severe pulmonary infection, is the most common causative agent [8,9].

The pathological processes resulting in ALI can be divided into inflammatory exudative, lung tissue proliferation, and lung fibrosis phases, although the pathogenesis is generally more complex than this. The Keap1/Nrf2 signaling pathway has been found to be associated with cancers such as breast, lung, and colon cancers, among others [10,11,12,13]. The Keap1/Nrf2/ARE pathway regulates the transcription of many antioxidants to maintain cellular homeostasis and prevent the organism from harm. Inflammation is considered to be an important factor leading to cancer, and the occurrence of inflammation is often accompanied by oxidative stress. It has been demonstrated [14] that nuclear factor erythroid 2-related factor 2 (Nrf2), a key factor in the cellular oxidative stress response, can ameliorate lung injury by inducing the expression of heme oxygenase-1 (HO-1) and NADPH quinone acceptor oxidoreductase 1 (NQO-1), which resist oxidative stress and responses induced by inflammation, for which the principal mediator is the Keap1/Nrf2/ARE pathway. Transduction of this pathway has therapeutic effects on both LPS-induced and sepsis-induced ALI [15,16]. It has also been demonstrated [17] that toll-like receptor (TLR) 4, expressed in significant concentrations on human platelets and megakaryocytes, is essential for the LPS-induced coagulation dysfunction and responses to inflammation observed in the lungs. Following activation of TLRs by LPS or sepsis in the respiratory system, high levels of pro-inflammatory mediators are produced, in combination with low levels of anti-inflammatory molecules, further launching the production of a cascade of downstream NF-κB and inflammatory factors, resulting in ALI [18,19].

It is generally accepted that Chinese medicines have fewer side effects than Western medicines, with various such medicines and experimental studies demonstrating good clinical efficacy for the prevention and treatment of ALI [20,21,22,23]. *Atractylodis rhizoma* has good anti-inflammatory and antioxidant effects, and also promotes epithelial cell barrier integrity [24,25], all of which are similar to the clinical pathological symptoms of ALI. However, the efficacy of *Atractylodis rhizoma* for the clinical treatment of ALI remains unknown, as do its possible mechanisms of action. Therefore, in the present study, an in-depth study of the efficacy of *Atractylodis rhizoma* for the treatment of ALI was conducted, and its mechanism of action investigated, in anticipation of defining the basis for the development of novel drugs to counteract ALI.

## 2. Results

### 2.1. HPLC Profile of the Chemical Composition of EEAR

**Figure 1:**
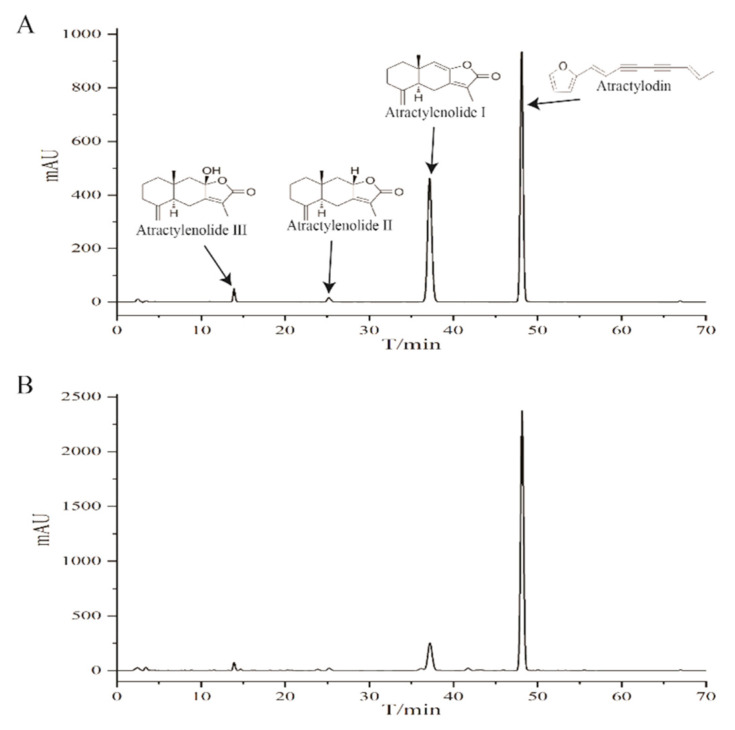
Chemical composition profile of EEAR. (**A**) Chromatogram of four mixed standards. (**B**) Chromatogram of EEAR.

As displayed in Figure 1, the chemical composition of the ethanolic extract of *Atractylodis rhizoma* (EEAR) was determined by high performance liquid chromatography (HPLC), with the identification of four components, namely, atractylenol, atractylenolide I, atractylenolide II, and atractylenolide III, of which atractylenol was in the highest concentration and atractylenolide I the second highest.

### 2.2. Effects of EEAR on Wet-Dry (W/D) Specific Gravity and the Inflammatory Response in the Lungs of Rats with ALI

**Figure 2:**
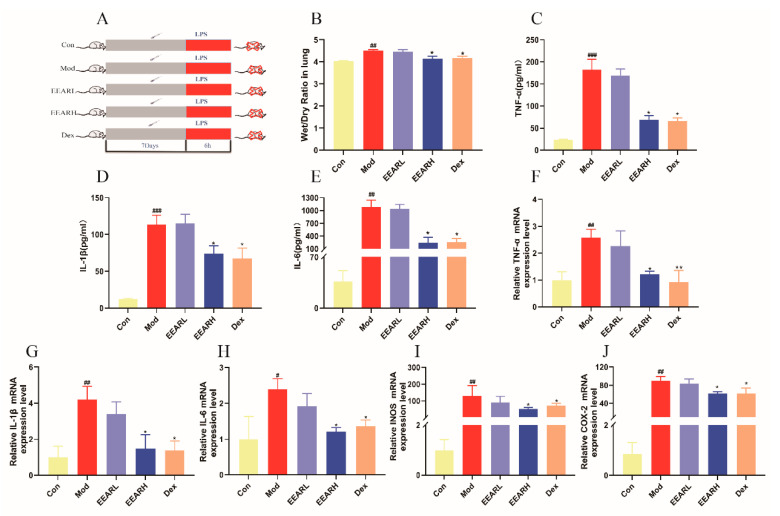
EEAR alleviated lung inflammation and reduced the wet-dry ratio in rats with ALI. (**A**) Experimental flowchart. (**B**) Lung *W*/*D* ratio determined 6 h after LPS challenge (*n* = 10). The levels of (**C**) TNF-α, (**D**) IL-10, and (**E**) IL-6 in BAFL were determined by ELISA (*n* = 10). The relative expression of (**F**) TNF-α, (**G**) IL-1β, (**H**) IL-6, (**I**) INOS, and (**J**) COX-2 in rat lung tissue were evaluated by RT-qPCR (*n* = 6). Values are expressed in means ± SEM. ^###^ *p* < 0.001 vs. Control; ^##^ *p* < 0.01 vs. Control; ^#^ *p* < 0.05 vs. Control; ** *p* < 0.01 vs. Model; * *p* < 0.05 vs. Model.

Since an inflammatory response is involved in the development and progression of ALI, inflammation in the lungs of rats with ALI was examined. As displayed in Figure 2B, the *W*/*D* ratio of the lung tissue was significantly higher in the model group than in the control group (*p* < 0.01). Compared with the model group, the *W*/*D* ratio of lung tissue in the high-dose EEAR (EEARH) and dexamethasone (Dex) groups was significantly lower (*p* < 0.05). Compared with the control group, the levels of TNF-α, IL-1β, and IL-6 in the bronchoalveolar lavage fluid (BALF) of rats in the model group were significantly higher (*p* < 0.01), and were significantly lower (*p* < 0.05) in the EEARH and Dex groups following EEAR and Dex treatments (Figure 2C–E). In addition, measurement by RT-qPCR of inflammatory factor mRNA expression in the lung tissue of rats with ALI demonstrated that the mRNA expression levels of TNF-α, IL-1β, IL-6, cyclooxygenase-2 (COX-2), and inducible nitric oxide synthase (INOS) were significantly higher in the model group than in the control group (*p* < 0.01), while the mRNA expression levels of TNF-α, IL-1β, IL-6, COX-2, and INOS were significantly lower in the EEARH and Dex groups after the administration of the drug treatments (*p* < 0.05) (Figure 2F–J).

### 2.3. Effects of EEAR on Neutrophils and Oxidative Stress Levels in the Lungs of Rats with ALI

**Figure 3:**
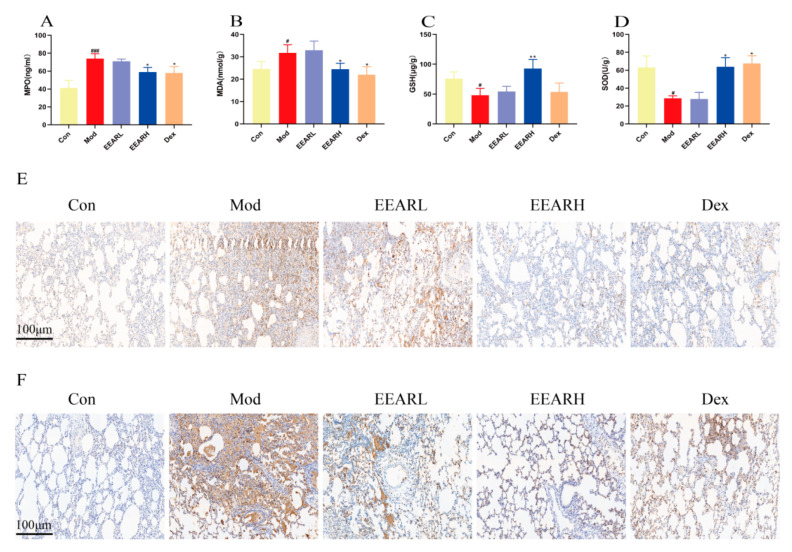
EEAR reduced neutrophil activation and oxidative stress levels in the lungs of rats with ALI. (**A**) MPO activity in the BAFL of rats (*n* = 10). (**B**) MDA activity in rat lung tissue (*n* = 10). (**C**) GSH activity in the lung tissue of rats (*n* = 10). (**D**) SOD activity in the lung tissue of rats (*n* = 10). (**E**) Immunohistochemical staining of MPO in lung tissue. (**F**) Immunohistochemistry showing neutrophils in lung tissue. Values are expressed as means ± SEM. ^###^ *p* < 0.001 vs. Control; ^#^ *p* < 0.05 vs. Control; ** *p* < 0.01 vs. Model; * *p* < 0.05 vs. Model.

ALI represents a pathological change due to pulmonary and systemic inflammatory responses. It has been demonstrated [26,27,28] that the production of lung inflammation in ALI is associated with excessive activation and aggregation of neutrophils in the lungs. Furthermore, excessive aggregation and activation of neutrophils can lead to the release of large quantities of reactive oxygen species (ROS) [29,30] which can further activate neutrophils and cause oxidative cellular damage, which aggravates the lung injury. Therefore, we examined neutrophil and oxidative stress levels in the lungs of rats with ALI. The results indicated that the level of myeloperoxidase (MPO) was significantly higher in the model group compared with the control group (*p* < 0.001), with a significant decrease (*p* < 0.05) in the EEARH and Dex groups after the administration of the respective drug treatments (Figure 3A). In terms of oxidative stress, the expression of malondialdehyde (MDA) was significantly higher, and the expression of superoxide dismutase (SOD) and glutathione (GSH) significantly lower, in the model group compared with the control group (*p* < 0.05), with a significant recovery in the EEARH and the Dex groups following the administration of the respective drug treatments (*p* < 0.05) (Figure 3B–D). In addition, immunohistochemistry demonstrated that the numbers of neutrophils and the concentrations of their expression product, MPO, were significantly elevated in the model group, with both decreasing significantly after administration of the drug treatments (Figure 3E,F).

### 2.4. Effect of EEAR on Lung Barrier Function in Rats with ALI

**Figure 4:**
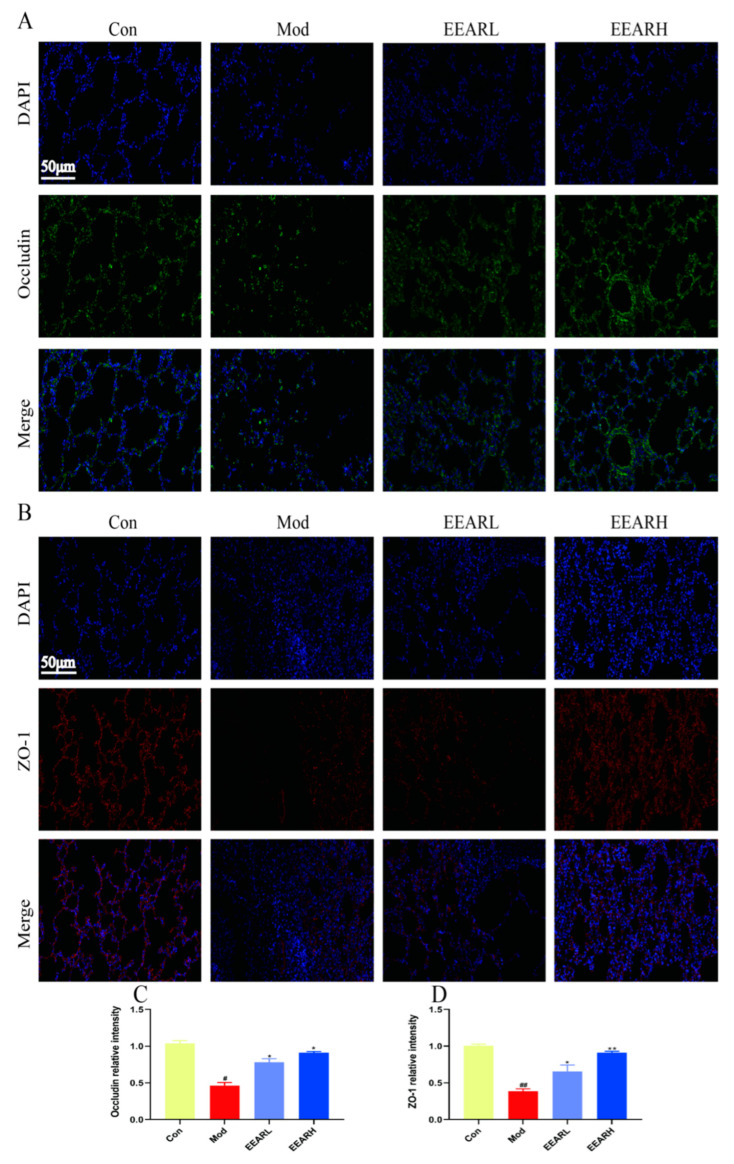
EEAR improved the lung epithelial barrier in rats with acute lung injury. (**A**) Immunofluorescence of the tight junction protein occludin in rat lung tissue. (**B**) Immunofluorescence of tight junction protein ZO-1 in rat lung tissue. (**C**) Plot of occludin levels (*n* = 3). (**D**) Plot of ZO-1 levels (*n* = 3). Values are expressed as means ± SEM. *^##^ p* < 0.01 vs. Control; *^#^ p* < 0.05 vs. Control; ** p < 0.01 vs. Model; * p < 0.05 vs. Model.

ALI is usually accompanied by damage to the alveolar-capillary barrier [31,32,33], and improving pulmonary epithelial barrier function is a principal means of treating ALI. Therefore, the barrier function of the lung epithelial cells was examined in the present study. The results demonstrated that the expression of the tight junction proteins zonula occluden-1 (ZO-1) and occludin were significantly reduced in the model group, with the lung barrier structure broken and significantly abnormal. There was significant recovery after the administration of EEAR, in which the effect of EEARH was superior to that of low-dose EEAR (EEARL) (Figure 4A–D). This indicates that EEAR reduced barrier dysfunction in the rat lungs and alleviated ALI.

### 2.5. Effects of EEAR on the TLR4/NF-κB Signaling Pathway

**Figure 5:**
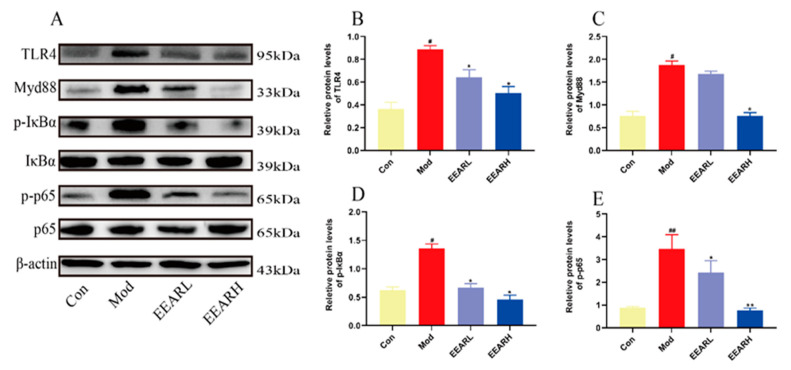
EEAR alleviated the extent of lung inflammation in rats with ALI by inhibition of the TLR4/NF-κB signaling pathway (*n* = 3). (**A**) TLR4/NF-κB signaling pathway expression levels in lung tissue measured by Western blot analysis. (**B**) Quantitative analysis of differences in TLR4 levels in each group. (**C**) Quantitative analysis of differences in Myd88 levels in each group. (**D**) Quantitative analysis of differences in p-IκBα levels in each group. (**E**) Quantitative analysis of differences in p-p65 levels in each group. Values are expressed as means ± SEM. *^##^ p* < 0.01 vs. Control; *^#^ p* < 0.05 vs. Control; ** p < 0.01 vs. Model; * p < 0.05 vs. Model.

LPS is a major component of the cell wall of Gram-negative bacteria and an important causative agent of inflammation [34]. It has been shown [35,36] that LPS-induced ALI is associated with the NF-κB signaling pathway, and in ALI, LPS is specifically recognized by TLR4, which activates the NF-κB signaling pathway to induce the release of multiple inflammatory factors, including TNF-α, IL-1β, and IL-6. We observed a significant increase in the expression of TLR4, Myeloid differentiation factor 88 (Myd88), phospho-IκB alpha (p-IκBα), and phospho-p65 (p-p65) in the model group using Western blot analysis of ALI rat lung tissue. Each sample demonstrated a different extent of decrease in expression after the administration of the EEAR treatment, with the greatest effect observed in the EEARH samples (Figure 5A–E).

### 2.6. Effects of EEAR on the Keap1/Nrf2 Signaling Pathway

**Figure 6:**
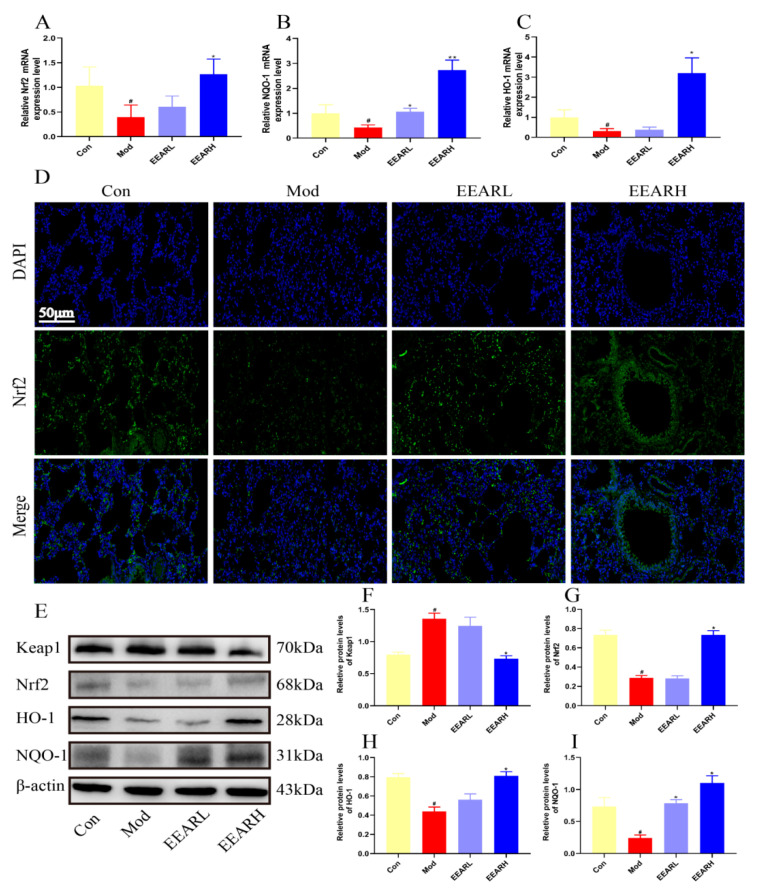
EEAR reduced oxidative stress levels in rats with ALI via the Keap1/Nrf2 signaling pathway. The relative expression of (**A**) Nrf2, (**B**) NQO-1, and (**C**) HO-1 in the lung tissue of rats was evaluated by RT-qPCR (*n* = 6). (**D**) Immunofluorescence of Nrf2 protein in rat lung tissue. (**E**) Keap1/Nrf2 signaling pathway expression levels in rat lung tissue were measured by Western blot analysis (*n* = 3). (**F**) Quantitative analysis of differences in Keap1 levels in each group (*n* = 3). (**G**) Quantitative analysis of differences in Nrf2 levels in each group (*n* = 3). (**H**) Quantitative analysis of differences in HO-1 levels in each group (*n* = 3). (**I**) Quantitative analysis of differences in NQO-1 levels in each group (*n* = 3). Values are expressed as means ± SEM. *^#^ p* < 0.05 vs. Control; ** p < 0.01 vs. Model; * p < 0.05 vs. Model.

Oxidative damage plays an important role in the development and pathology of ALI [37]. The relationship between antioxidants and ALI has received increasing attention over recent years, including study of the Keap1/Nrf2 signaling pathway. It has been shown [38,39] that this pathway is able to mitigate the extent of ALI by inhibiting the activation of NF-κB and affecting the release of inflammatory factors in vivo. In addition, the Keap1/Nrf2 signaling pathway, when activated during periods of oxidative stress, promotes the expression of the downstream protein HO-1, thereby alleviating the level of oxidative stress in the host [40]. By examining the lung tissue in rats with ALI, we found that the Nrf2 mRNA expression levels and those of its downstream targets, NQO-1 and HO-1, were significantly lower in the model group (*p* < 0.05), while EEAR promoted Nrf2 mRNA expression levels and those of its downstream targets (*p* < 0.05) (Figure 6A–C). The immunofluorescence plots also indicated a significant decrease in Nrf2 expression in the model group and a significant increase following the administration of EEAR (Figure 6D). Finally, the Keap1/Nrf2 signaling pathway was examined by Western blot analysis. The results indicate that the expression of Keap1 was significantly higher in the model group, while the expression of Nrf2, HO-1, and NQO-1 was significantly lower, an effect reversed by the administration of EEAR (Figure 6E–I).

### 2.7. Effect of EEAR on LPS-Induced Inflammatory Factor Expression in THP-1 Cells In Vitro

**Figure 7:**
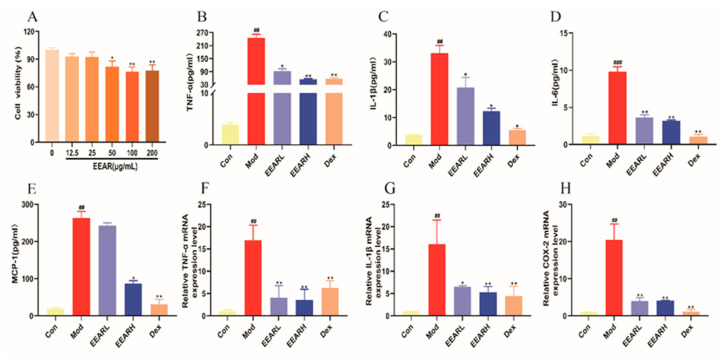
EEAR inhibited the expression of LPS-induced inflammatory factors in THP-1 cells. (**A**) Cell viability after EEAR exposure measured using a CCK-8 assay (*n* = 5). (**B**) Expression of TNF-α in cell supernatants (*n* = 3). (**C**) Expression of IL-1β in cell supernatants (*n* = 3). (**D**) Expression of IL-6 in cell supernatants (*n* = 3). (**E**) Expression of MCP-1 in cell supernatants (*n* = 3). Relative expression of (**F**) TNF-α, (**G**) IL-1β, and (**H**) COX-2 in LPS-stimulated THP-1 cells evaluated by RT-qPCR (*n* = 3). Values are expressed as means ± SEM. *^###^ p* < 0.001 vs. Control; *^##^ p* < 0.01 vs. Control; ** p < 0.01 vs. Model; * p < 0.05 vs. Model.

To further confirm the anti-inflammatory effect of EEAR, LPS-stimulated THP-1 cells were used to simulate an in vitro model of ALI. Firstly, the required EEAR dose was confirmed by CCK-8 assay, with 12.5 μg/mL utilized as the low-dose group and 25 μg/mL as the high-dose group (Figure 7A). Secondly, inflammatory factor expression was measured in the cell supernatants. The expression of TNF-α, IL-1β, IL-6, and Monocyte chemotactic protein-1 (MCP-1) were each significantly higher in the model group, and significantly lower after treatment with EEAR (Figure 7B–E). In addition, these observations were reflected in the RT-qPCR results (Figure 7F–H).

### 2.8. Effects of EEAR on the TLR4/NF-κB Signaling Pathway In Vitro

**Figure 8:**
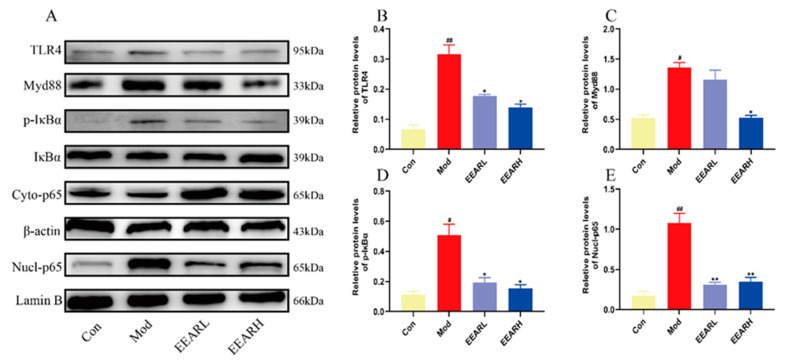
EEAR inhibited LPS-induced activation of the TLR4/NF-κB signaling pathway in THP-1 cells (*n* = 3). (**A**) TLR4/NF-κB signaling pathway expression levels in cells as measured by Western blot analysis. (**B**) Quantitative analysis of differences in TLR4 levels in each group. (**C**) Quantitative analysis of differences in Myd88 levels in each group. (**D**) Quantitative analysis of differences in P-IκBα levels in each group. (**E**) Quantitative analysis of differences in nuclear p65 levels in each group. *^##^ p* < 0.01 vs. Control; *^#^ p* < 0.05 vs. Control; ** p < 0.01 vs. Model; * p < 0.05 vs. Model.

The effects of EEAR on the TLR4/NF-κB signaling pathway were confirmed using additional in vitro experiments. The results suggest that the expression of TLR4, Myd88, and p-IκBα were significantly higher in the model group than in the control group. It was also found that the expression of p65 in the nucleus was significantly increased. The expression of each protein was lower to varying degrees following treatment with EEAR (Figure 8A–E). These results suggest that EEAR also inhibits activation of the TLR4/NF-κB signaling pathway in vitro, consistent with the results of the in vivo experiments.

### 2.9. Effects of EEAR on the Keap1/Nrf2 Signaling Pathway In Vitro

**Figure 9:**
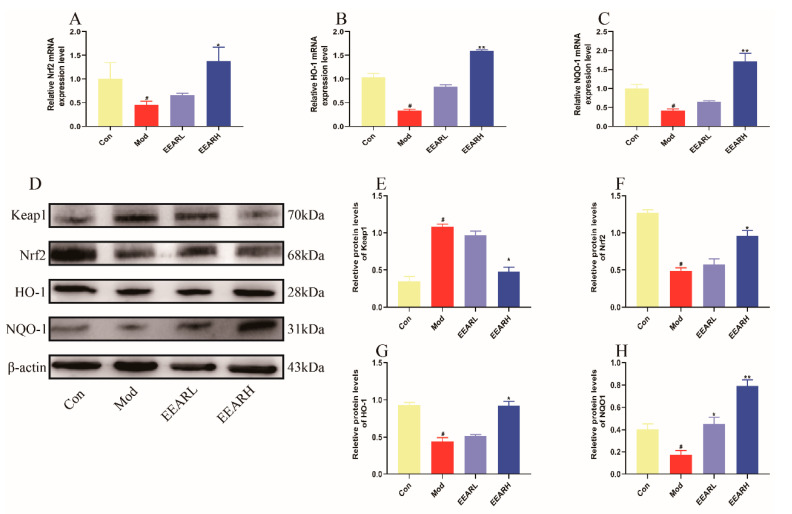
EEAR alleviated LPS-induced oxidative stress in THP-1 cells via the Keap1/Nrf2 signaling pathway (*n* = 3). Relative (**A**) Nrf2, (**B**) HO-1, and (**C**) NQO-1 expression in cells was evaluated by RT-qPCR. (**D**) Keap1/Nrf2 signaling pathway expression levels in cells were measured by Western blot analysis. (**E**) Quantitative analysis of differences in Keap1 levels in each group. (**F**) Quantitative analysis of differences in Nrf2 levels in each group. (**G**) Quantitative analysis of differences in HO-1 levels in each group. (**H**) Quantitative analysis of differences in NQO-1 levels in each group. Values are expressed as means ± SEM. *^#^ p* < 0.05 vs. Control; ** p < 0.01 vs. Model; * p < 0.05 vs. Model.

Whether EEAR was able to reduce LPS-induced oxidative stress in THP-1 cells via the Keap1/Nrf2 signaling pathway was further clarified in in vitro experiments. RT-qPCR results indicate that the expression of Nrf2, HO-1, and NQO-1 was significantly lower in the model group than in the control group (*p* < 0.05), but significantly higher following administration of EEAR (*p* < 0.01) (Figure 9A–C). In addition, Western blot analysis demonstrated that in the model group, the expression of Keap1 protein was significantly higher, while the expression of the antioxidant protein Nrf2 and its downstream targets, NQO-1 and HO-1, were significantly lower. These observations were reversed after treatment with EEAR. This indicates that EEAR also alleviated LPS-induced oxidative stress in THP-1 cells in vitro via the Keap1/Nrf2 signaling pathway, consistent with the results of the in vivo experiments.

## 3. Discussion

ALI is a syndrome characterized by an excessive inflammatory response causing dyspnea and hypoxemia due to damage in the alveolar epithelial cells and capillary endothelial cells caused by a variety of injury-causing factors, both inside and outside the lung. It is a critical condition causing high morbidity and mortality [41,42]. The pathogenesis of ALI can be divided into three principal phases: increased alveolar–capillary membrane permeability, imbalance in the inflammatory and anti-inflammatory responses, and changes in the pulmonary and bronchial circulation [43,44]. Currently, clinical treatments for ALI commonly include pharmacotherapy and mechanical ventilation [45,46,47]. Although ventilator treatment strategies can provide a degree of relief to patients, they are not effective in reducing the morbidity and mortality rate, while conventional Western drugs such as dexamethasone can cause adverse side effects, including coagulation dysfunction, gastric ulcers, and osteoporosis. Chinese herbal medicines are generally considered clinically effective in preventing and treating ALI, with a number of experimental studies of Chinese herbal medicines demonstrating beneficial therapeutic effects. This provides the rationale for researching and developing other herbal medicines for the prevention and treatment of ALI.

*Atractylodis rhizoma* is a traditional Chinese medicine which has previously been shown [48] to exhibit excellent anti-inflammatory properties and to provide protection of epithelial cell barrier function, both aspects lacking in the pathogenesis of acute lung injury. Therefore, one could speculate that *Atractylodis rhizoma* may represent a potential candidate for the treatment of ALI.

LPS is a pathogenic endotoxin of the outer membrane of Gram-negative bacteria and is widely used to establish animal models of ALI [49]. In the present study, a rat model of ALI was established by tracheal drip injection of LPS. Lung tissue examination of the model group indicated that the lung tissue *W*/*D* ratio was significantly higher than in other groups, with correspondingly higher levels of the inflammatory factors TNF-α, IL-6, and IL-1β in BALF, with increased numbers of neutrophils, indicating that the model had been successfully replicated. EEAR significantly reduced the level of inflammatory factors and neutrophilia in rat BALF, decreased the lung *W*/*D* ratio, reduced pulmonary edema, decreased levels of MDA in rat lung tissue, and significantly increased GSH and SOD activity. The results demonstrate that EEAR significantly protected against LPS-induced lung inflammatory injury.

A number of herbal medicines have been found to be effective for treating ALI [50,51], with their mechanisms of action occurring through three possible pathways: an anti-inflammatory response, anti-oxidative stress, and protection of airway epithelial barrier function. Numerous studies have concluded [52,53,54] that the TLR4/NF-κB pathway is capable of rapidly initiating intracellular inflammatory signaling pathways in ALI in response to LPS. The binding of TLR4 to LPS can activate multiple signaling pathways, the principal pathway being the MyD88 pathway, which activates IκB kinase (IKK) after a cascade of signals, followed by NF-κB inhibitor (IκB) phosphorylation, and the shedding of IκB from NF-κB which is subsequently ubiquitinated. NF-κB thus becomes activated from its inhibited state and then rapidly translocates to the nucleus, binding to its target protein and swiftly upregulating the expression of pro-inflammatory cytokines such as IL-1β, TNF-α, IL-6, and IL-8. In the present study, EEAR was demonstrated to reduce the expression of inflammatory factors in the lungs of rats with ALI in both in vivo and ex vivo experiments via inhibition of the TLR4/NF-κB signaling pathway, thereby alleviating the inflammatory response to ALI.

LPS induces the aggregation and hyperactivation of inflammatory cells, which generates a large degree of ROS-induced oxidative stress. Antioxidation is a key strategy for treating ALI [55]. The Keap1/Nrf2 signaling pathway is an important pathway that inhibits oxidative stress. Keap1 and Nrf2 are normally bound and in an inactive state. When stimulated in some way, their binding becomes unstable, releasing Nrf2 which binds to the antioxidant responsive element (ARE), which regulates the transcription of a variety of downstream antioxidant proteins and metabolic enzyme genes, thereby inhibiting the oxidative stress response in vivo. NQO-1 and HO-1, downstream target genes of the Keap1/Nrf2 signaling pathway, have anti-inflammatory, antioxidant, and anti-apoptotic properties [56,57]. In the present study, we found that EEAR inhibited the expression of Keap1 and promoted the expression of Nrf2 and its downstream proteins NQO-1 and HO-1, thus reducing the level of oxidative stress in acute lung injury.

**Figure 10:**
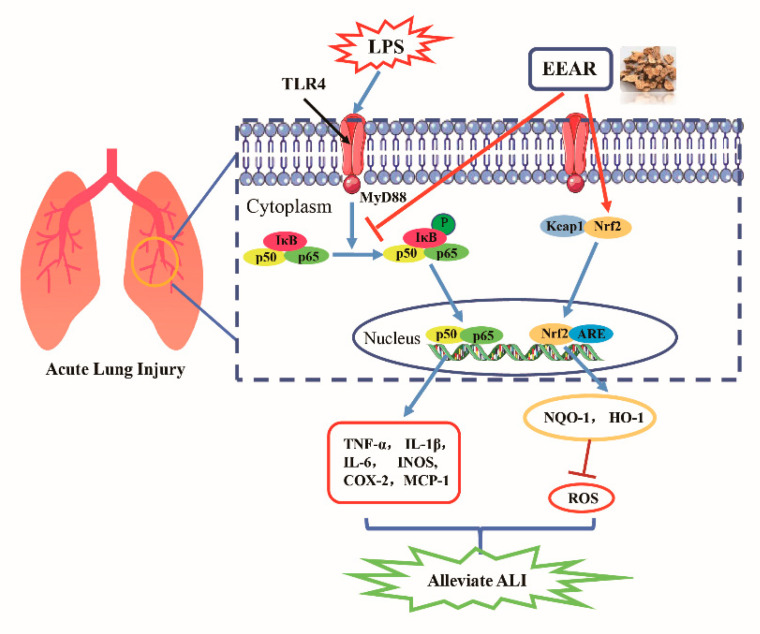
EEAR attenuates oxidative stress and inflammatory responses via TLR4/NF-κB and Keap1/Nrf2 signaling pathways to alleviate LPS-induced ALI.

Over recent years, with the rapid development of molecular biology and cell biology research techniques, experimental investigation of the prevention and treatment of ALI using Chinese medicines has made significant progress. However, research on their mechanisms of action is limited, and mostly at the level of identification of inflammatory factors, oxidative stress, and inflammatory cell infiltration. In-depth research and exploration remain somewhat uncommon. In the present study, we have only provided a preliminary exploration of the efficacy and mechanism of action of EEAR against ALI. It is clear that EEAR reduces the symptoms of ALI via the TLR4/NF-κB and Keap1/Nrf2 signaling pathways (Figure 10), but additional study of the mechanisms of action is required, including using inhibitors and knockout rats. In addition, *Atractylodis rhizoma* has multiple chemical components and identification of the factor or factors that assist in the prevention and treatment of ALI requires additional research.

## 4. Materials and Methods

### 4.1. Extract of Atractylodis rhizoma

*Atractylodis rhizoma* was obtained from the Hubei Tianji Chinese Herbal Pieces Company (Wuhan, China). The authenticity of the medicinal materials was confirmed by Kun Yu, an associate Professor from the College of Pharmacy of Hubei University of Chinese Medicine. A total of 200 g of *Atractylodis rhizoma* was crushed and then passed through a No. 2 sieve, soaked overnight in a 10-fold greater volume of 80% ethanol, then extracted 3 times in an ultrasonic water bath for 2 h each time, then filtered, after which the extracts were combined and concentrated in a rotary evaporator. The residue was dried under vacuum and weighed, from which the yield of volatile alcoholic extract was calculated.

### 4.2. Conversion of Dosage

The Chinese Pharmacopoeia (2020 Edition) lists the clinical dosage of *Rhizoma atractylodis* as 3–9 g, with 9 g representing the dose for an adult having a standard weight of 60 kg. Thus, the daily adult dose is 150 mg/kg. Using an accepted dose conversion between humans and rats, the dosage given to rats should be 925.5 mg/kg. Thus, a low dose was defined as 925.5 mg/kg and a high dose as 7404.0 mg/kg [25]. Based on the yield in an 80% ethanol extract of *Rhizoma atractylodis* (34%), the dosage of the ethanol extract of *Rhizoma atractylodis* in rats ranged from 314.7 mg/kg to 2517.4 mg/kg. 

### 4.3. Sample Preparation and HPLC Chromatographic Conditions

A 0.5 g quantity of EEAR powder was placed into a 100 mL conical flask with a stopper, to which methanol was added and extracted in an ultrasonic water bath, then cooled to 25 °C. The samples were shaken well and allowed to stand, after which methanol was added to adjust the weight, then centrifuged at 4 °C at 5000× *g* for 10 min. The supernatant was filtered through a 0.22 µm membrane and stored until required for further use.

An appropriate quantity of atractylodin, atractylenolide I, atractylenolide II, and atractylenolide III were weighed and dissolved in methanol to yield a 1 mg/mL stock solution. Atractylodin (purity > 98%), atractylenolide I (purity > 98%), atractylenolide II (purity > 98%), and atractylenolide III (purity > 98%) were purchased from Chengdu Push Biotechnology Co., Ltd. (Chengdu, China).

HPLC was performed using an Agilent 1260 Infinity system. Chromatographic separation was achieved using an Agilent C18 column (250 mm × 4.6 mm; 5 µm) at 25 °C. The eluent was acetonitrile (A)–aqueous formic acid (100:0.1, *v*/*v*) (B) at a flow rate of 1.0 mL/min using an injection volume of 10 µL. Gradient elution was performed as follows: 50% A: 0–10 min; 55% A: 10–35 min; 65% A: 35–45 min; 72% A: 45–48 min; 85% A: 48–70 min.

### 4.4. In Vivo Experimental Design

Sterile-pathogen-free (SPF) male Sprague Dawley (SD) rats (140 ± 20 g) were purchased from the experimental animal center of Three Gorges University (Yichang, China; animal license No. SCXK (E) 2022–0012). The rats were maintained in an animal room at a temperature of 24 ± 1 °C and humidity of 50–70% with a 12 h/12 h light/dark cycle and adaptive feeding for a week. The husbandry and all surgical procedures on the experimental animals met all ethical requirements and were approved by the animal ethics committee of the Hubei University of Chinese Medicine (approval code: NO. 00273292, 10 November 2020). 

The SD rats (*n* = 60) were randomly divided into the following five groups, 12 rats per group: normal group, model group, EEARL group, EEARH group, and Dex group. The ethanol extract of Atractylodis Rhizome was diluted with normal saline to prepare 31.47 mg/mL and 251.74 mg/mL solutions. Dexamethasone was dissolved in normal saline to prepare a 0.5 mg/mL solution. On days 1 to 7, a daily volume of normal saline (1 mL/100 g) was administered to the normal and model groups by gavage, and the corresponding drug concentrations to the other groups by gavage (1 mL/100 g). LPS (5 mg/kg) was administered to all groups other than the normal group two hours after gavage on the seventh day by intratracheal instillation. After 6 h, the animals were anesthetized using pentobarbital sodium. Subsequently, lung tissue samples and BALF were collected and evaluated using immunohistochemistry (IHC), immunofluorescence, Western blot analysis, and enzyme-linked immunosorbent assays (ELISAs).

### 4.5. Lung Wet/Dry Ratios

The whole lungs were removed, washed with PBS solution three times, and then the water on the surface of the lungs was dried with absorbent paper and weighed immediately, giving the wet weight. Then they were put into the incubator, baked at 80 °C for 48 h, and then weighed again, giving the dry weight, and then the ratio of wet weight to dry weight (*W*/*D*) was calculated.

Wet/Dry Ratio in lung = wet weight/dry weight

### 4.6. ELISA Assays

BALF obtained from each rat was centrifuged and the supernatants collected for the measurement of MPO (RK03821), TNF-α (RK00029), IL-6 (RK00020), and IL-1β (RK00009) secretion using enzyme-linked immunosorbent assay (ELISA) kits, in accordance with the manufacturer’s instructions. ELISA kits used to analyze in vivo samples were purchased from ABclonal Technology Co., Ltd. (Wuhan, China). In vitro analysis was conducted by seeding *THP*-1 cells (2 × 10^5^ cells/well) in 6-well plates and incubating with phorbol myristate acetate (PMA, 100 ng/mL) for 24 h, followed by the addition of LPS (1 μg/mL), IFN-γ (20 ng/mL), and EEAR (12.5 μg/mL and 25 μg/mL) or Dex (10 μmol/mL) for 24 h, following removal of the supernatant. Cell-free supernatants were collected for analysis of the secretion of MCP-1 (E-EL-H6005), TNF-α (E-EL-H0109c), IL-6 (E-EL-H6156), and IL-1β (E-EL-H0149c). All ELISA kits for the analysis of cellular secretion were purchased from Elabscience Biotechnology Co., Ltd. (Wuhan, China). PMA (P1585), LPS (L2880), IFN-γ (SER3058), and Dex (D4902) were purchased from Sigma-Aldrich, St. Louis, MO, USA.

### 4.7. Measurement of MDA, GSH, and SOD Levels in Lung Tissue

Extracted lung tissue was homogenized and dissolved in extraction buffer, then analyzed for MDA (BC0025), SOD (BC0175), and GSH (BC1175) concentration using commercially available assay kits purchased from Beijing Solarbio Science & Technology Co., Ltd. (Beijing, China), in accordance with the manufacturer’s instructions. MDA concentration provided an evaluation of the extent of lipid peroxidation in the lung tissue. SOD and GSH levels provided measures of antioxidative enzyme activity. 

### 4.8. Immunohistochemical Staining

For IHC, lung tissues were fixed in a 4% (*w*/*v*) solution of paraformaldehyde in PBS overnight, rinsed with PBS, and stored in 70% (*v*/*v*) ethanol. The samples were embedded in paraffin and 5 μm sections were obtained. Once deparaffinized, the sections were processed for antigen retrieval by incubation in 10 mM sodium citrate buffer (pH 6.0) containing 0.05% (*v*/*v*) Tween 20 at 95 °C for 10 min, washed twice with 0.1% (*v*/*v*) Triton X-100 in PBS, blocked for 45 min in 2% (*v*/*v*) donkey serum in 0.1% (*v*/*v*) Triton X-100 in M PBS, and incubated overnight at 4 °C with primary antibody (1:200) for MPO (Abcam, Cambridge, UK, ab208670), Ly-6 g (Abcam, Cambridge, UK, ab238132). The sections were washed in PBS and incubated for 2 h at room temperature with secondary antibody (1:500, Servicebio Technology Co., Ltd. Wuhan, China).

### 4.9. Immunofluorescence

Immunostaining was performed in accordance with previously published protocols [58] using primary antibodies for ZO-1 (1:500, Abcam, ab216880), occludin (1:500, Abcam, ab167161), and Nrf2 (1:500, Proteintech, Wuhan, China, 16396-1-AP). After incubation with the corresponding secondary antibody (1:1000), cell nuclei were stained with DAPI (Roche, Shanghai, China) for 15 min, after which fluorescence microscopy was used for analysis and image acquisition (Olympus, Tokyo, Japan).

### 4.10. RNA Extraction and RT-qPCR

Total RNA in THP-1 cells or lung tissue was extracted using Trizol reagent (Vazyme Biotech Co., Ltd., Nanjing, China), in accordance with the manufacturer’s instructions. RNA purity was determined from the A_260nm_/A_280nm_ absorption ratio and was considered acceptable at a value between 1.8 and 2.0. The RNA was reverse-transcribed into cDNA using a commercial kit (Vazyme Biotech Co., Ltd., Nanjing, China). Primers were synthesized by the Tsingke Biotechnology Co., Ltd. RT-qPCR was performed using a ChamQ SYBR qPCR Master Mix (Vazyme Biotech Co., Ltd., Nanjing, China) in a Roche Light Cycler 96 system (Roche, Basel, Switzerland). The PCR reaction was conducted as follows: 95 °C for 30 s, then 40 cycles at 95 °C for 10 s, and 60 °C for 30 s. Melt curve analysis was conducted after exposure of the samples to 95 °C for 15 s, 60 °C for 60 s, and 95 °C for 15 s. All gene expression values were normalized to β-actin expression and calculated using the 2^−∆∆CT^ method [59]. The specific primer sequences for all genes quantified are listed in Table 1.

### 4.11. Cell Culture and CCK-8 Assay

THP-1 cells (China Cell Line Bank, Beijing, China) were cultured in RPMI 1640 medium supplemented with 10% fetal bovine serum (FBS, Gibco, Grand Island, NY, USA), and 100 U/mL penicillin and 100 U/mL streptomycin at 37 °C in a humidified atmosphere containing 5% CO_2_. In all experiments, the cells were allowed to acclimate for 24 h prior to any treatment. Analysis was conducted in accordance with a previous study [60]. The viability of THP-1 macrophages following the various treatments was measured using a CCK-8 cell proliferation and cytotoxicity assay kit (CA1210; Beijing Solarbio Science & Technology Co., Ltd., Beijing, China). Briefly, THP-1 cells were seeded in 10% FBS-RPMI at a density of 5 × 10^4^ cells/100 μL in 96-well plates, after which the cells were treated with PMA (100 ng/mL) and EEAR (0, 12.5, 25, 50, 100, 200 μg/mL) for 24 h. A 10 µL CCK-8 solution was added to each well and incubated at 37 °C for 1.5 h, in accordance with the manufacturer’s instructions, after which the absorbance at 450 nm was measured using a microplate reader (Thermo Fisher Scientific, Waltham, MA, USA).

### 4.12. Western Blot Analysis

Lung tissue samples or cells were lysed in radioimmunoprecipitation assay (RIPA) buffer containing protease and phosphatase inhibitors, for 30 min. Total protein concentration was measured using a bicinchoninic acid (BCA) protein assay kit (Elabscience Biotechnology Co., Ltd. Wuhan, China). A total of 20 μg of protein solution was electrophoretically transferred onto a PVDF membrane following separation on a 10% SDS-polyacrylamide gel. The membrane was blocked using 5% (*w*/*v*) nonfat dry milk blocking solution for 1 h, followed by overnight incubation at 4 °C with the respective primary antibody (1:1000). The membrane was then incubated for an additional hour with the appropriate HRP-conjugated secondary antibody (1:5000 dilution) at room temperature after thoroughly washing three times with phosphate buffered saline containing Tween (PBST). Bands were detected by enhanced chemiluminescence (ECL, Amersham Pharmacia Biotech, Piscataway, NJ, USA) and band intensities quantified using Image J software [61]. The proteins Myd88 (4283S), IκBα (4814S), p-IκBα (2859S), p65 (8242S), HO-1 (43966S), p-p65 (3033S), and β-actin (3700S) were purchased from Cell Signaling Technology, Inc. (Danvers, MA, USA). TLR4 (66350-1-Ig), Keap1 (1053-2-AP), NQO-1 (67240-1-Ig), and LaminB1 (12987-1-AP) were purchased from Proteintech Group, Inc. (Wuhan, China).

### 4.13. Isolation of Nuclear and Cytosolic Fractions

Cytoplasmic and nuclear extracts were prepared using an NE-PER nuclear and cytoplasmic extraction reagent kit (Beijing Solarbio Science & Technology Co., Ltd., Beijing, China), in accordance with the manufacturer’s instructions. All steps were performed on ice, or at 4 °C.

### 4.14. Statistical Analysis

The results are presented as means ± S.E.M. All data were analyzed using GraphPad Prism version 8.0 software (San Diego, CA, USA). A Student’s *t*-test or one-way ANOVA followed by Bonferroni test was used to compare two independent variables: ^#^ *p* < 0.05, ^##^ *p* < 0.01, * *p* < 0.05, ** *p* < 0.01.

## 5. Conclusions

The results indicate that EEAR was effective in reducing the pathological symptoms of ALI, alleviating pulmonary edema, and improving the lung barrier. EEAR not only reduced the secretion of inflammatory factors in the lung by inhibition of TLR4/NF-κB signaling pathway activation, but also reduced the oxidative stress response in the lung via the Keap1/Nrf2 signaling pathway, thus alleviating ALI. The results help to clarify the efficacy of EEAR in preventing and treating ALI, as well as to clarify its mechanism of action. They provide the basis for the further development of therapeutic drugs for ALI.

## Figures and Tables

**Table 1:** Primer sequences used in RT-qPCR.

Name	Primer Sequences (from 5′→3′)	
Rat-TNF-α	Forward	GGAGGGAGAACAGCAACTCC
	Reverse	GCCAGTGTATGAGAGGGACG
Rat-IL-1β	Forward	AGGCTGACAGACCCCAAAAG
	Reverse	GGTCGTCATCATCCCACGAG
Rat-IL-6	Forward	AGAGACTTCCAGCCAGTTGC
	Reverse	AGTCTCCTCTCCGGACTTGT
Rat-INOS	Forward	GGGGACTGGACTTTTAGAGACG
	Reverse	CCGTGGGGCTTGTAGTTGAC
Rat-COX-2	Forward	GTTCATCCCGGATCCCCAAG
	Reverse	ACGTGGGGAGGGTAGATCAT
Rat-Nrf2	Forward	GGTTGCCCACATTCCCAAAC
	Reverse	CAGGGCAAGCGACTGAAATG
Rat-NQO-1	Forward	CGGCTCCATGTACTCTCTGC
	Reverse	GAGTGGTGACTCCTCCCAGA
Rat-HO-1	Forward	GCCTGGTTCAAGATACTACCTCT
	Reverse	CTGAGTGTGAGGACCCATCG
Rat-β-actin	Forward	GCAGGAGTACGATGAGTCCG
	Reverse	ACGCAGCTCAGTAACAGTCC
Human-TNF-α	Forward	TCTTCTCGAACCCCGAGTGA
	Reverse	TATCTCTCAGCTCCACGCCA
Human-IL-1β	Forward	GGCTGCTCTGGGATTCTCTT
	Reverse	ATTTCACTGGCGAGCTCAGG
Human-COX-2	Forward	TGCTGGTGGAAAAACCTCGT
	Reverse	AAAACCCACTTCGCCTCCAA
Human-Nrf2	Forward	GATCTTGGAGTTGCCCACATTC
	Reverse	CAAGTGACTGAAACGTAGCCG
Human-NOQ-1	Forward	TCCCCCTGCAGTGGTTTG
	Reverse	CATGTCCCCGTGGATCCCTT
Human-HO-1	Forward	CTCCGGCAGTCAACGCCT
	Reverse	CTCTGACAAATCCTGGGGCA
Human-β-actin	Forward	GGATTCCTATGTGGGCGACGA
	Reverse	GCGTACAGGGATAGCACAGC

## Data Availability

The data presented in this study are available in the article.
